# Club Activity, Study Groups, and Academic Achievement: A Nationwide Study of Japanese Medical Students' Extracurricular Life

**DOI:** 10.1002/jgf2.70118

**Published:** 2026-04-13

**Authors:** Hirohisa Fujikawa, Hidetaka Tamune, Yuji Nishizaki, Hirotake Mori, Sho Fukui, Kiyoshi Shikino, Taro Shimizu, Yu Yamamoto, Hiroyuki Kobayashi, Toshio Naito, Yasuharu Tokuda

**Affiliations:** ^1^ Department of General Medicine Juntendo University Faculty of Medicine Bunkyo‐ku Tokyo Japan; ^2^ Department of Medical Education Studies, International Research Center for Medical Education, Graduate School of Medicine The University of Tokyo Bunkyo‐ku Tokyo Japan; ^3^ Center for General Medicine Education, School of Medicine Keio University Shinjuku‐ku Tokyo Japan; ^4^ Department of Psychiatry and Behavioral Science Juntendo University Graduate School of Medicine Bunkyo‐ku Tokyo Japan; ^5^ Division of Medical Education Juntendo University School of Medicine Bunkyo‐ku Tokyo Japan; ^6^ Department of Emergency and General Medicine Kyorin University Mitaka Tokyo Japan; ^7^ Division of Rheumatology, Inflammation, and Immunity Brigham and Women's Hospital and Harvard Medical School Boston Massachusetts USA; ^8^ Department of Community‐Oriented Medical Education, Graduate School of Medicine Chiba University Chiba Chiba Japan; ^9^ Department of Diagnostic and Generalist Medicine Dokkyo Medical University Hospital Mibu Tochigi Japan; ^10^ Division of General Medicine, Center for Community Medicine Jichi Medical University Shimotsuke Tochigi Japan; ^11^ Department of Internal Medicine Mito Kyodo General Hospital, University of Tsukuba Mito Ibaraki Japan; ^12^ Muribushi Okinawa for Teaching Hospitals Urasoe Okinawa Japan; ^13^ Tokyo Foundation for Policy Research Minato‐ku Tokyo Japan

**Keywords:** academic achievement, clinical knowledge, club activity, medical knowledge, study group, undergraduate medical education

## Abstract

**Background:**

Student involvement in extracurricular activities (ECAs) possesses multiple benefits and has attracted substantial interest in medical education. Most studies on ECAs have been conducted in Western contexts, and findings on the association between ECAs and academic achievement are mixed. We examined the current status of ECA, particularly club activities and study groups, the most representative ECAs in Japan, among medical trainees.

**Methods:**

This was a nationwide cross‐sectional study from April to May 2025 in Japan, using an online anonymous self‐administered questionnaire. Potential participants were takers of the General Medicine In‐Training Examination postgraduate “Year‐0” (GM‐ITE PGY‐0). We asked the participants about their participation in club activities and study groups during their medical school life and evaluated their medical knowledge using their GM‐ITE PGY‐0 test score. The data were analyzed by descriptive statistics and multivariable linear regression analysis with adjustment for possible confounders.

**Results:**

We enrolled 437 of 748 medical trainees. 398 (91.1%) were involved in club activities, predominantly those involving music, while 156 (35.7%) participated in study groups, with clinical reasoning emerging as the most popular. On multivariable linear regression analysis, weekly or more frequent participation in club activities was associated with lower medical knowledge compared with no participation. In contrast, study group participation was positively and dose‐dependently associated with medical knowledge test score.

**Conclusions:**

Medical schools should consider strategies to encourage academic ECAs such as study groups while promoting balanced engagement with nonacademic ECAs to optimize both their potential benefits and learning outcomes.

## 1 Introduction

Extracurricular activities (ECAs) have been a topic of interest in medical education over the past few decades [1]. ECA is defined as an academic or nonacademic activity that is performed under the auspices of the school but which occurs outside of regular classroom time and not as part of the curriculum [2, 3, 4]. Participation in ECAs can facilitate personal development that is challenging to nurture in regular classroom time only [5]. The heavy academic load and stress of medical education make the journey to completion long and emotionally taxing. ECA likely serves as a buffer against this stress and the potential burnout it can produce [6, 7, 8]. Moreover, participation in ECAs has the potential to foster leadership skills [9], and contributes to enhancing communication skills and self‐efficacy [10, 11]. Student involvement in ECAs is thus a crucial component of medical education and has many benefits.

ECA involves a range of activities. Although no agreed categorization has been established, it is commonly divided into two groups: direct ECAs and indirect ECAs [8]. Whereas direct ECAs are regarded as “one that is more closely associated with the student's major or curriculum,” indirect ECAs are defined as “one that is relatively unrelated to the student's major or curriculum.” [2] Indirect ECAs are composed of subcategories, such as prosocial activities, artistic activities, team sports, self‐governance or entertainment clubs, academic clubs unrelated to the regular curriculum, research activities, and career development [8].

Prior studies on ECA are hampered by three major issues. First, most were conducted in Western countries [8]. Despite the likelihood that student engagement in ECA is affected by cultural and/or historical background, this has yet to be fully examined in the Eastern context. Second, although several studies have examined the link between ECA participation and academic achievement, their findings are mixed: some studies demonstrated a negative association between student engagement in ECA and academic achievement, consistent with the time‐displacement explanation commonly discussed in ECA research, whereby increased participation reduces opportunities for deliberate study [4, 12]. Other studies have shown that ECA participation is positively linked with academic achievement, possibly reflecting a developmental framework in which students acquire cognitive or motivational benefits indirectly through ECA involvement [4, 5, 13, 14]. Third, medical education has been affected by the coronavirus disease 2019 (COVID‐19) pandemic [15, 16]. The worldwide promotion of social distancing to reduce the spread of infection in the acute phase of the pandemic resulted in increased adoption of an online lifestyle, which affected student engagement in ECAs [17]. Although the level of threat has since decreased, the pandemic remains a concern and is likely to have impacted ECA engagement. Thus, examining the current status of medical students' ECAs in Japan as of 2025 is of high priority.

Notably, Japan's ECA ecology differs significantly from the typical Western system, which makes international comparisons essential. Within medical schools, *bukatsudo* (club activities) represents the most traditional and institutionally supported form of ECA, with the majority of students participating under the belief that such involvement fosters social development and personal growth [18]. In parallel, study groups are also common and function as a culturally embedded form of peer‐assisted learning. Study groups in Japan are more likely to remain informal and autonomously organized by students, with no institutional coordination, whereas some Western medical schools have introduced formally structured peer‐assisted learning programs [19]. Participation in either club activities or study groups can be time‐intensive; yet despite their cultural importance, there is little comprehensive data on engagement patterns and their impact on academic achievement among medical trainees in Japan.

Accordingly, we aimed to clarify the current status of ECA, particularly club activities and study groups, among medical students in Japan as of 2025. We anticipated that the findings of this study would deepen our knowledge of ECAs.

## 2 Methods

### 2.1 Design, Setting, and Participants

We performed this nationwide cross‐sectional study from April 4 to May 31, 2025, in Japan. It comprised a part of a series of research projects on student engagement and extracurricular activities in undergraduate medical education. We asked the examinees of the General Medicine In‐Training Examination postgraduate “Year‐0” (GM‐ITE PGY‐0) to participate in the study following the examination. Prior to participation, they were informed that participation was voluntary and that nonparticipation would not result in any disadvantage. We included only participants who agreed to participate in the present study. As the aim of the study was to clarify the current status of ECA among medical students in Japan, we excluded participants who had graduated from medical schools outside Japan.

In the Japanese medical education system, medical trainees usually graduate from university in March and initiate early residency programs from April [20]. The timing of this study thus allowed the study participants to reflect on their life during their university years immediately after graduation [21]. The GM‐ITE PGY‐0 was developed in 2018 by the Japan Institute for Advancement of Medical Education Program (JAMEP, a nonprofit organization) to assess medical knowledge at the beginning of residency [21]. The examination is now taken by many medical trainees across Japan [22, 23, 24].

### 2.2 Measures

We developed a web‐based questionnaire that asked participants about their ECAs during their medical school, with reference to previous studies [5, 25, 26]. We asked them about their participation in club activities and study groups and their frequency and content. Regarding content, multiple answers were allowed.

We assessed the participants' medical knowledge by the test scores of the GM‐ITE PGY‐0. The GM‐ITE was developed using a methodology similar to that of the U.S. Residency Internal Medicine In‐Training Examination. It has been validated for its ability to assess levels of medical knowledge [27, 28]. The scores range from 0 to 80, with higher scores indicating greater clinical knowledge. We selected covariates for their potential association with ECAs and medical knowledge. We included age, sex, location of university, and type of university.

### 2.3 Data Analysis

We computed descriptive statistics. Continuous variables were reported as means and standard deviation, while categorical variables were reported as counts and percentages. For questions allowing multiple responses (i.e., the content of ECAs), percentages were calculated using the number of respondents to each question as the denominator.

To elucidate the association between ECAs and medical knowledge, we used multivariable linear regression analysis. The intraclass correlation coefficient for the outcome variable was computed, yielding a value below 0.1. This finding indicated that the clustering effect based on the alma mater medical school was negligible [29]. Accordingly, we determined that multilevel analysis was not necessary and that conventional analysis (multivariable linear regression analysis) would suffice [29]. A two‐sided *p*‐value < 0.05 was considered statistically significant. We utilized SPSS version 29.0.2.0 (IBM Corp) for all statistical analysis.

### 2.4 Ethical Considerations

All participants expressed their agreement to participate in the study by checking a consent box in the online questionnaire. Ethical approval was obtained from the ethics committee of the JAMEP (no. 24–22).

## 3 Results

Of the possible 748 participants, 471 provided informed consent to participate. Of these, we excluded 12 individuals from foreign medical schools. Of the remaining 459 participants, we then excluded 22 with missing data. Accordingly, 437 were included in the analysis. Table 1 shows the participants' profiles.

**TABLE 1:** Characteristics of the participants (*N* = 437).

Characteristics	*N* (%)
Sex	
Female	143 (32.7)
Male	294 (67.3)
Age	
24	173 (39.6)
25	137 (31.4)
26–30	105 (24.0)
≥ 31	22 (5.0)
Type of university	
National or public university	281 (64.3)
Private university	156 (35.7)
Location of university	
Rural	281 (64.3)
Urban	156 (35.7)
GM‐ITE PGY‐0 score (range: 0–80), mean (SD)	42.82 (7.02)

Abbreviations: GM‐ITE PGY‐0, General Medicine In‐Training Examination postgraduate “Year‐0”; SD, standard deviation.

Table 2 demonstrates the frequency of club activities and study groups during medical school. 398 (91.1%) participated in club activities. Of these, the majority participated once a week or more (339, 77.6%). Regarding study groups, 281 (64.3%) did not participate in study groups at all, whereas the remaining 156 (35.7%) did participate, of whom 116 (26.5%) participated in a study group for less than once a week.

**TABLE 2:** Frequency of club activities and study groups (*N* = 437).

	*N* (%)
Club activities	
Did not participate at all	39 (8.9)
Less than once a week	59 (13.5)
Once a week or more	339 (77.6)
Study group	
Did not participate at all	281 (64.3)
Less than once a week	116 (26.5)
Once a week or more	40 (9.2)

Table 3 shows the content of the club activities and study groups. Three hundred eight provided responses regarding the content of their club activities. 274 (89.0%) and 78 (25.3%) participated in sporting and nonsporting club activities, respectively. Popular club activities included music (46, 14.9%), tennis (27, 8.8%), golf (25, 8.1%), badminton (20, 6.5%), and table tennis (20, 6.5%). Fifty‐eight provided responses to the content of their study group. The most popular study group was about clinical reasoning (16, 27.6%), followed by national medical license examination preparation (9, 15.5%).

**TABLE 3:** Content of the club activities and study groups.

Club activities^a^	
Content	*N* (%)
Sporting clubs	
Tennis	27 (8.8)
Golf	25 (8.1)
Badminton	20 (6.5)
Table tennis	20 (6.5)
Swimming	18 (5.8)
Baseball	17 (5.5)
Kyudo (Japanese archery)	16 (5.2)
Volleyball	16 (5.2)
Soccer	15 (4.9)
Basketball	13 (4.2)
Track and field	12 (3.9)
Dance	10 (3.2)
Rowing	9 (2.9)
Karate	7 (2.3)
Futsal	6 (1.9)
Handball	6 (1.9)
Skiing	6 (1.9)
Rugby	5 (1.6)
American football	4 (1.3)
Kendo	4 (1.3)
Archery	3 (1.0)
Ice hockey	3 (1.0)
Judo	3 (1.0)
Mountaineering	3 (1.0)
Aikido	2 (0.6)
Sailing	2 (0.6)
Softball	2 (0.6)
Subtotal	274 (89.0)
Nonsporting clubs	
Music^c^	46 (14.9)
Medical studies	13 (4.2)
Japanese tea ceremony	5 (1.6)
Photography	2 (0.6)
Volunteer	2 (0.6)
Art	1 (0.3)
Automobiles	1 (0.3)
Calligraphy	1 (0.3)
Fishing	1 (0.3)
Gardening	1 (0.3)
Shogi	1 (0.3)
Others	4 (1.3)
Subtotal	78 (25.3)

Study group^b^	
Content	*N* (%)
Clinical reasoning	16 (27.6)
National medical license examination preparation	9 (15.5)
Clinical procedure skill	5 (8.6)
Emergency and intensive care	4 (6.9)
English for medical purposes	4 (6.9)
Physiological examination	3 (5.2)
Community medicine	2 (3.4)
Family medicine	2 (3.4)
Pediatrics	2 (3.4)
Research	2 (3.4)
Surgery	2 (3.4)
Internal medicine	1 (1.7)
Physical examination	1 (1.7)
Others	10 (17.2)

^a^ 308 provided responses. Participants can provide multiple answers. Percentages were calculated with a denominator of 308.

^b^ 58 provided responses. Participants can provide multiple answers. Percentages were calculated with a denominator of 58.

^c^ Music‐related clubs included classical ensemble‐based activities (e.g., orchestra, choir, and chamber music; *n* = 23) and band‐based activities (*n* = 22); one response did not specify the type.

To examine the association of these ECAs with medical knowledge, we performed multivariable linear regression analysis (Table 4). We noted a trend indicating that participation in club activities once a week or more was associated with lower medical knowledge test scores (adjusted mean difference, −2.98; 95% confidence interval, −5.28 to −0.67, compared with the no participation group). Conversely, participation in a study group showed a dose‐dependent positive association with medical knowledge test scores (e.g., adjusted mean difference, 3.19; 95% confidence interval, 0.94 to 5.45 for participation once a week or more, compared with the no participation group).

**TABLE 4:** Association between club activity, study group, and medical knowledge^a^.

	Unadjusted mean difference (95% confidence interval)	Adjusted^b^ mean difference (95% confidence interval)
Club activities		
Did not participate at all	Ref.	Ref.
Less than once a week	−0.78 (−3.61 to 2.06)	−1.45 (−4.21 to 1.32)
Once a week or more	−2.41 (−4.73 to −0.09)*	−2.98 (−5.28 to −0.67)*
Study group		
Did not participate at all	Ref.	Ref.
Less than once a week	1.91 (0.41 to 3.42)*	1.99 (0.52 to 3.46)**
Once a week or more	2.74 (0.43 to 5.05)*	3.19 (0.94 to 5.45)**

^a^ Medical knowledge test scores ranged from 0 to 80, with higher scores indicating greater medical knowledge.

^b^ Adjusted for age, sex, university location, and university type.

*p < 0.05, **p < 0.01.

## 4 Discussion

In this study, we examined the current status of participation in club activities and study groups, which we deemed the most representative ECAs in Japanese medical schools. The study produced three notable findings. First, a considerable number of medical students joined in club activities or study groups. In particular, more than 90% of participants reported participation in club activities. Second, descriptive statistics revealed the most popular ECA: music in club activities and clinical reasoning in study groups. Third, multivariable linear regression analyses demonstrated that frequent participation in club activities tended to be associated with lower medical knowledge test scores, whereas participation in study groups might have a potentially positive association with greater medical knowledge. These findings offer novel insights into the complex role of ECAs in medical education.

The high prevalence of club activity participation among medical students is consistent with a previous study in Japan [18]. That study elucidated membership of clubs by medical students in only a single medical school in Japan and found that approximately 90% of all medical students were involved in club activities [18]. These findings likely reflect the cultural emphasis placed on group‐based extracurricular involvement in Japan. In addition, the result of our nationwide study that the majority of participants joined in music club activities was noteworthy. This agrees with a study by Ledger and Joynes in the U.K., who demonstrated that medical students at their medical school had high levels of engagement and experience with music, and that music was interwoven with medical students' lives [30]. However, we note that most of the school life of our study participants coincided with the acute phase of the coronavirus disease 2019 (COVID‐19) pandemic, which would have had an impact on their ECAs. For example, social distancing was particularly emphasized in Japan to reduce COVID‐19 transmission [31], which likely led to less participation in contact sports. Future studies should examine the ECAs of the medical students who entered their medical school after the acute phase of the pandemic.

To our knowledge, this study is the first to examine the current status of study group participation among medical students. We found that approximately one‐third of the respondents reported involvement in a study group, with clinical reasoning emerging as the predominant theme. Recognition of the necessity for explicit and systematic teaching of clinical reasoning is increasing across the world, due to its potential to enhance patient safety and improve quality of healthcare delivery [32, 33]. In Japan, however, the enhancement of clinical reasoning skills among medical trainees mainly depends on individual motivation and experience, and opportunities for systematic education are limited [34]. Accordingly, Japanese students with high motivation to learn may participate in clinical reasoning study groups, while those with less motivation may refrain. Potential barriers, such as growing formal curricular demands of medical school and limited time due to other forms of ECA (e.g., part‐time jobs), can inhibit study group participation.

Furthermore, we made the noteworthy finding that there may be contrasting relationships between club activities and academic performance, and between study groups and academic performance. Specifically, while frequent involvement in club activities tended to be associated with less medical knowledge, study group participation might be positively associated with greater medical knowledge test scores. This finding is consistent with a recent study in Thailand, which showed a positive linear correlation between the frequency of organizing educational ECAs and grade point average, and a curvilinear relationship between the frequency of organizing recreational and miscellaneous ECAs and grade point average [5]. However, the Thai study was limited in several regards. First, it was a single‐center study, which raises concerns about generalizability; and second, it was a retrospective correlational study and thus could not control for potential confounders. Conversely, our present study collected nationwide data via the JAMEP network and adjusted for possible confounders. Thus, our study contributes to the literature and provides valuable insights into ECA in medical students' lives.

Given these significant relationships between club activities, study groups, and academic performance, we suggest two strategies. First, considering that frequent participation might be associated with diminished academic performance, we propose that medical schools should monitor the frequency of students' club activities. Nevertheless, this proposal should be considered vis a vis a growing body of evidence indicating that indirect ECAs, including sports and music, are highly advantageous for medical trainees: a Canadian study reported that sports appeared to be associated with higher motivation and well‐being among medical students [35]; Lodewyk et al. [36] also demonstrated that participation in individual sports may have a significant association with greater tolerance for ambiguity and uncertainty; and Fares et al. [7] showed that music‐related activities were significantly correlated with low burnout. Accordingly, any consideration of club activities should contemplate both the benefits and potential adverse consequences arising from excessive engagement. Given that participation in club activities less than once a week in our study was not significantly associated with greater medical knowledge test scores than no participation, medical schools should facilitate club activities while monitoring the level of engagement. Second, medical schools should support the involvement of students in study groups. Only one‐third of medical students participated in the study groups, suggesting that there may have been potentially motivated medical students who were interested in participating but were unable to do so. It may be beneficial for medical schools to provide support for such students.

Finally, we list several potential limitations of this study. First, a degree of social desirability bias may be present. We attempted to minimize this concern to the extent possible, for example by conducting the survey anonymously. Second, because participation in the GM‐ITE PGY‐0 was voluntary, the study population may have represented a selected subgroup of motivated medical trainees, introducing selection bias. For example, highly motivated trainees without prior ECA participation may have devoted more time to academic study or exam preparation. Restricting the analysis to these selected participants may therefore have biased the observed association. Future studies including a broader and more representative sample of trainees are warranted. Third, because of the cross‐sectional design, causal relationships cannot be inferred. In particular, reverse causality cannot be ruled out; for example, trainees with greater medical knowledge may have been more likely to participate in study groups rather than study group participation improving knowledge, and trainees with lower academic achievement or less study‐oriented attitudes may have been more likely to engage frequently in club activities. Fourth, medical knowledge was assessed after the initiation of residency. The timing of the survey would have been ideal if it had been conducted just before the medical students graduated from medical school. However, a number of important events occur at or near the time of graduation, including the national medical license examination and graduation ceremony [21]. These events prevent the survey from being conducted before graduation. Nevertheless, we conducted this study immediately following the start of the residency program, which allowed us to capture the medical knowledge obtained during medical school life in a relatively concise manner. Fifth, the questionnaire used in this study has not been validated. However, it was developed with reference to previous studies [5, 25, 26]. Future studies should examine its reliability and validity. Sixth, although music‐related clubs and clinical reasoning–focused study groups were common, the present study did not examine the educational implications of specific activity content. Future research may clarify how different types of ECAs contribute to professional development. Seventh, as described above, the participants led most of their medical student life during the acute phase of the COVID‐19 pandemic. This may have affected student engagement in ECAs, particularly contact sports. Eighth, this study was conducted in a single Asian country. Student involvement in ECAs is likely affected by cultural and/or historical background. A comparison of these findings with those of similar studies outside Asia would enable a more profound understanding of the benefits and harms associated with ECAs.

## 5 Conclusions

This study found that a substantial number of medical students participated in club activities or study groups. Over 90% joined in club activities, with the most popular being music. Approximately one‐third reported participation in a study group, with clinical reasoning emerging as the predominant topic. Additionally, we found that while excessive involvement in club activities might have a negative association with lower medical knowledge test scores, study group participation may be associated with greater medical knowledge. With regard to club activities, it is important to consider both the well‐evidenced benefits (e.g., greater well‐being, higher motivation, and enhanced ambiguity tolerance) and potential adverse consequences arising from excessive engagement. This study further suggests that medical schools should promote and facilitate the participation of students in study groups. Participation may be hindered by various factors, including increasing demands of the formal curricular and the limited time available due to frequent engagement in other forms of ECAs, however, and we therefore recommend that medical schools provide support for these students.

## Author Contributions

**Hidetaka Tamune:** conceptualization, investigation, methodology, writing – review and editing, supervision, formal analysis support. **Hiroyuki Kobayashi:** conceptualization, investigation, methodology, writing – review and editing, funding acquisition, formal analysis support, supervision. **Yu Yamamoto:** conceptualization, investigation, methodology, writing – review and editing, formal analysis support, supervision. **Hirohisa Fujikawa:** conceptualization, investigation, writing – original draft, methodology, software, formal analysis, project administration, data curation, funding acquisition. **Taro Shimizu:** conceptualization, investigation, methodology, writing – review and editing, formal analysis support, supervision. **Sho Fukui:** conceptualization, investigation, methodology, writing – review and editing, formal analysis support, supervision. **Toshio Naito:** conceptualization, investigation, methodology, writing – review and editing, formal analysis support, supervision. **Hirotake Mori:** conceptualization, investigation, methodology, writing – review and editing, supervision, formal analysis support. **Yuji Nishizaki:** conceptualization, investigation, methodology, writing – review and editing, supervision, formal analysis support. **Kiyoshi Shikino:** conceptualization, investigation, methodology, writing – review and editing, formal analysis support, supervision. **Yasuharu Tokuda:** conceptualization, investigation, methodology, writing – review and editing, formal analysis support, supervision.

## Funding

This work was partly supported by the Health, Labour and Welfare Policy Grants of Research on Region Medical (no. 24IA2016) from Japan's Ministry of Health, Labour and Welfare.

## Disclosure

Other information: Y.N. is an Editorial Board member of the Journal of General and Family Medicine. To minimize bias, Y.N. was excluded from all editorial decision‐making related to the acceptance of this article for publication.

## Ethics Statement

The ethics committee of the JAMEP approved this study (no. 24–22).

## Consent

Prior to participating in the study, all study participants provided informed consent in the online questionnaire.

## Conflicts of Interest

H.T., S.F., K.S., T.S., and Y.Y. have received honoraria from Japan Institute for Advancement of Medical Education Program (JAMEP) as exam preparers of the General Medicine In‐Training Examination (GM‐ITE). Y.N. has received an honorarium from JAMEP as GM‐ITE project manager. K.S. and H.K. have received honoraria from JAMEP as speakers at JAMEP lectures. Y.T., the director of JAMEP, has received an honorarium from JAMEP as a speaker at a JAMEP lecture. H.T., Y.N., S.F., K.S., T.S., Y.Y., H.K., and Y.T. were not involved in the data analysis. The other authors declare no conflicts of interest.

## Data Availability

The data that support the findings of this study are available on request from the corresponding author. The data are not publicly available due to privacy or ethical restrictions.
